# Advances in Celiac Disease and Gluten-Free Diet

**DOI:** 10.3390/nu14030570

**Published:** 2022-01-28

**Authors:** Isabel Comino, Carolina Sousa

**Affiliations:** Department of Microbiology and Parasitology, Faculty of Pharmacy, University of Seville, 41012 Seville, Spain; csoumar@us.es

Celiac disease (CD) is a systemic disease that causes chronic enteropathy of the small intestine and develops through an inadequate immune response to gluten in genetically predisposed individuals [1,2]. To date, the only effective treatment is a gluten-free diet (GFD) that essentially relies on the consumption of naturally gluten-free foods and gluten-free dietary products that may not contain more than 20 mg/kg of gluten according to Codex Alimentarius (Codex Standard 118-19792). Research on CD is changing rapidly due to a steady increase in knowledge that addresses its pathophysiology, diagnosis, follow-up, and therapeutic options. For this reason, this Special Issue includes 12 peer-reviewed articles reporting on the latest research findings and evidence related to CD and a GFD.

CD is characterized by a heterogeneous clinical presentation, affecting any organ or tissue with gastrointestinal, extraintestinal, seronegative, or nonresponsive manifestations. A common, and sometimes the only, clinical finding in untreated patients is anemia, which is generally caused by damage of the duodenal mucosa and the resulting iron malabsorption. However, a poor correlation has been found between the presence of anemia, an abnormal expression of duodenal iron transport proteins, and the severity of histological damage [3]. In other cases, the onset of EC is represented by subclinical manifestations, some of which can be found in the mouth. In this Special Issue, Nota et al. [4] report significant associations between the clinical characteristics of CD and the prevalence of caries and dentin sensitivity. Additionally, an inappropriate GFD was associated with oral manifestations.

Regarding extraintestinal manifestations, CD has been associated with IgA nephropathy (IgAN), the most common primary chronic glomerular disease worldwide. Furthermore, it is well known that IgA-class tissue transglutaminase (tTG) autoantibodies are deposited in the small intestine mucosa and extraintestinal organs. Nurmi et al. [5] have identified IgA deposits targeted for tTG in kidney biopsies of gluten-consuming IgAN patients with or without known CD.

The diagnosis of CD is based on several criteria, including positive serology, a spectrum of duodenal damage, clinical symptoms and/or risk conditions, and response to a GFD in patients with HLA-DQ2 or DQ8 genotypes. In the absence of some of these criteria, especially when serology is negative or duodenal atrophy is incomplete, the diagnosis of CD becomes challenging. Ruiz-Ramírez et al. [6] have confirmed the high diagnostic accuracy of the intraepithelial lymphocyte cytometric pattern as a tool in the diagnosis of CD regardless of the degree of mucosal damage and age.

As Viitasalo et al. [7] proposed, close relatives of patients with CD, with partially shared living environments and genetic factors, could have increased seroreactivity to microbial markers. They studied the seropositivity and levels of anti-*Saccharomyces cerevisiae* (ASCA), *Pseudomonas fluorescens*-associated sequence (anti-I2), and *Bacteroides caccae* TonB-linked outer membrane protein (anti-OmpW) in first-degree relatives and patients with CD. The results showed an increase in seroreactivity to serum microbial markers, particularly ASCA and anti-I2, in relatives of patients with CD, even in the absence of disease-specific autoantibodies or other signs of active CD. This observation was not explained by the presence or absence of predisposing HLA haplotypes, suggesting the role of other genetic and environmental factors.

Since the only effective treatment for CD is a GFD, several patients have difficulty controlling their diet, especially those who live in a rural setting [8] and, therefore, regularly consume sufficient gluten to trigger symptoms. In their reviews, Weiser et al. [9,10] discuss gluten contamination and adherence to a GFD. The available evidence on the degree of adherence to a GFD, barriers to its implementation [9,10], and methods to assess it [11] were examined. Despite the availability of diverse traditional GFD adherence markers, such as diet tests or serology, none of them are an accurate diet evaluation method. Thus, the use of gluten immunogenic peptides (GIP) detection in urine has been developed as a direct test for GFD monitoring, contrary to classical methods. Coto et al. provide new knowledge on gluten metabolism and GIP excretion in urine [12].

Segura et al. [13] discuss emerging therapeutic options for CD based on the removal of toxic gluten peptides, the modulation of intestinal permeability, or the restoration of the gut microbiota. These treatment options have shown encouraging preliminary results in phase II and III clinical trials. If successful, these novel approaches raise the possibility of reintroducing gluten, in amounts to be determined, into the diets of patients with CD. However, a GFD is the mainstay of CD therapy for the immediate future, pending FDA and/or EMA approval of any of these treatment options.

Finally, a GFD has been evaluated in other gastrointestinal pathologies. Several trials have evaluated the ‘bottom-up’ approach of a GFD in irritable bowel syndrome (IBS), with a response rate between 34 and 71%. Fernández-Bañares et al. [14] studied the effect of a GFD in patients with functional bowel disorders (FBD) and evaluated the role of both the low-grade celiac score and the celiac lymphogram in the probability of responding to a GFD. Their study shows that a GFD is effective in the long-term treatment of patients with previously unexplained chronic watery diarrhea or dominant bloating symptoms that meet the criteria for FBD. The response rate was much higher in the subgroup of patients defined by the presence of a positive low-grade celiac score and celiac lymphogram.

In conclusion, these valuable studies provide a deeper understanding of the diagnosis, follow-up of patients with EC, and the effect of a GFD. We thank all the authors for their contributions, the reviewers for their constructive comments, and the Nutrients Publishing Team for their professional assistance in the development of this Special Issue.
